# Challenges in Achieving Viral Suppression Among Adolescents and Young Adults Under a Dolutegravir-Based Regimen: Living with HIV in Gabon

**DOI:** 10.3390/pathogens15050502

**Published:** 2026-05-07

**Authors:** Berthold Bivigou-Mboumba, Pamela Moussavou-Boundzanga, Falone L. Akombi, Carine Eyi Zang, Aurore F. Bouassa-Bouassa, Sahara Luzolo, Pélagie Okome, Augustin Mouinga-Ondeme, Simon Ategbo

**Affiliations:** 1 Unité Mixte de Recherche CIRMF-SSM, Centre Interdisciplinaire de Recherches Médicales de Franceville, Libreville BP 8507, Gabon; djiafafa@yahoo.fr (F.L.A.); aurorefayeb@gmail.com (A.F.B.-B.); 2 Département de Biologie, Université des Sciences et Technique de Masuku (USTM), Franceville BP 901, Gabon; pamelamoussavoub@gmail.com; 3 Service de Pédiatrie, Centre Hospitalier Universitaire Mère-Enfant Fondation Jeanne Ebori, Rue Pierre Barro, Libreville BP 2254, Gabon; eyizang@yahoo.fr (C.E.Z.); sategbo@yahoo.fr (S.A.); 4 Centre de Traitement Ambulatoire Pédiatrique, Centre Hospitalier Universitaire de Libreville, 38 Rue Docteur KONG, Libreville BP 2228, Gabon; luzolosarah27@yaoo.com; 5 Service de Pédiatrie, Hôpital d’Instruction des Armées Omar Bongo Ondimba (HIA-OBO), Libreville BP 20404, Gabon; okomepelagie@yahoo.fr; 6 Unité des Infections Rétrovirales et Pathologies Associées, Centre Interdisciplinaire de Recherches Médicales de Franceville, Franceville BP 769, Gabon; ondeme@yahoo.fr

**Keywords:** HIV, children, adolescents, young adults, viral suppression, Gabon

## Abstract

Background: Children, adolescents and young adults living with HIV represent a vulnerable population. Achieving viral suppression in this population remains a major challenge in sub-Saharan Africa. Methods: We conducted a cross-sectional study among HIV-positive individuals aged 0–24 years in Gabon. Data were collected, including viral load (VL), CD4 counts, and immunosuppression levels. Viral suppression was defined as VL < 1000 copies/mL. Statistical comparisons across age groups and immunosuppression categories were performed. Results: Of the 130 (100%) participants included, 59 (45.4%) were males and 71 (54.6%) females. Overall, 72 (55.4%) achieved viral suppression, while 58 (44.6%) remained uncontrolled. Viral suppression increased with age (23.8% (5/21) in 0–7 years; 29.2% (14/48) in 8–15 year and 42.6% (26/61) in 16–24 years), yet uncontrolled VL were predominant across all groups. Median VL values were low but showed wide interquartile ranges, indicating heterogeneity in viral control. Mean CD4 counts declined significantly with age (780 in 0–8 years vs. 470 in 16–24 years; *p* = 0.001). Immunosuppression levels correlated inversely with virological control (*p* < 0.001). Conclusions: Despite moderate overall viral suppression, nearly half of adolescents and young adults failed to achieve virological control. The decline in CD4 counts with age highlights increased vulnerability in this population.

## 1. Introduction

The global burden of HIV among children and adolescents remains significant, with an estimated 2.8 million individuals under the age of 20 living with HIV.

In sub-Saharan Africa, approximately 88% of children live with HIV, 83% of new pediatric HIV infections, and 84% of AIDS-related child deaths occur, underscoring the region’s disproportionate burden [1,2]. In these regions, pediatric HIV care is often challenged by delayed diagnosis, limited access to virological monitoring, and suboptimal adherence, particularly during adolescence [3,4,5]. The actual treatment is a Dolutegravir (DTG)-based regimen, which is considered the preferred agent due to its higher barrier to resistance, potent antiviral activity, once-daily dosing, and improved tolerability compared to non-nucleoside reverse transcriptase inhibitors (NNRTIs) and protease inhibitors (PIs) [5,6,7]. Early observational data from Tanzania, Lesotho, and Cameroon indicate virological suppression rates above 80% after 12 months of DTG-based ART in children and adolescents [2,7]. However, adolescents remain particularly vulnerable to virological failure, due to psychosocial factors, transitional gaps in care, and limited adherence support [6,7]. These challenges contribute to suboptimal progress toward the UNAIDS 95–95–95 targets, which aim to ensure that 95% of all people on ART achieve sustained viral suppression by 2030.

In Gabon, the HIV prevalence among the general population is estimated at 3.4%, with a younger demographic increasingly involved in HIV care programs [8]. Despite the national adoption of DTG-based ART regimens for children, adolescents and young adults since 2020, data on their real-world effectiveness remain limited. This study aims to describe age-stratified virological and immunological profiles among children (0 to 8 years), adolescents (9 to 16 years), and young adults (17 to 24 years) living with HIV on DTG-based ART in Gabon, and to discuss implications for age-tailored care and programmatic interventions.

## 2. Materials and Methods

We conducted a cross-sectional, observational study from January to September 2023 in Libreville, Gabon. Participants were recruited from three major reference centres for pediatric and adolescent HIV care: the paediatrics department, Jeanne Ebori Mother and Child University Hospital Centre; Outpatient Treatment Centre, Libreville University Hospital Centre and department; Omar Bongo Ondimba Military Teaching Hospital (HIA-OBO).

We included HIV-infected individuals aged from 1 to 24 years (children from 0 to 11 months of age were considered as 1 year old) on first-line ART for at least nine months.

The CD4 lymphocyte count measurements were performed using the BD FACSPresto System (BD Biosciences, San Jose, CA, USA), according to the manufacturer’s instructions. The HIV-1 viral load testing was performed using the GeneXpert HIV-1 Quant assay on the GeneXpert System (Cepheid, Sunnyvale, CA, USA). The detection threshold was 40 copies/mL. Sociodemographic and clinical data were extracted from patient records. All data were anonymised and compiled into Microsoft Excel (2013 Version) for analysis.

Data were entered into an Excel spreadsheet and analysed using STATA 14 software. Qualitative characteristics were described in terms of frequency (percentage) and quantitative variables in terms of mean (±standard deviation). Age was categorised into three groups (0–8, 8–16, 16–24 years). The immunity level variable was transformed into three categorical variables: highly immunocompromised (patients with a CD4 cell count under 200 cells/mm^3^); weakly immunocompromised (patients with a CD4 range between 200 and 500 cells/mm^3^), and immunocompetent (patients with a CD4 cell count at 500 cells/mm^3^ upper). For virological analysis, we defined the following analytic categories: “suppressed” (VL < 40 copies/mL), “controlled” (40 ≤ VL < 1000 copies/mL), and “uncontrolled” (VL ≥ 1000 copies/mL). The viral load values were transformed into log10. Normality of quantitative variables was assessed using a histogram and a normal plot. Comparison of proportions was performed using Pearson’s chi-2 test or Fisher’s exact test when expected frequencies were less than 5. The Kruskal–Wallis test was used to compare quantitative variables (CD4 counts and VL). The Pearson correlation test was used to assess the association between CD4 counts and viral loads. *p* values < 0.05 were considered statistically significant.

## 3. Results

### 3.1. Study Population Composition

A total of 130 children, adolescent and young adult were recruited for this study. The cohort comprised 59 (45.4%) males and 71 (54.6%) females. Among them, 125 (96.2%) were on Dolutegravir-based regimens, and 5 (3.8%) were on ABC-3TC-LPV/r. The most representative age span for the cohort was 16 to 24 years old (46.9%; 61/130), followed by 8 to 15 years old (36.9%; 48/130), and finally 0 to 7 years old (16.2%; 21/130) (Table 1).

### 3.2. Patient Immunity and Age Groups

**Figure 1:**
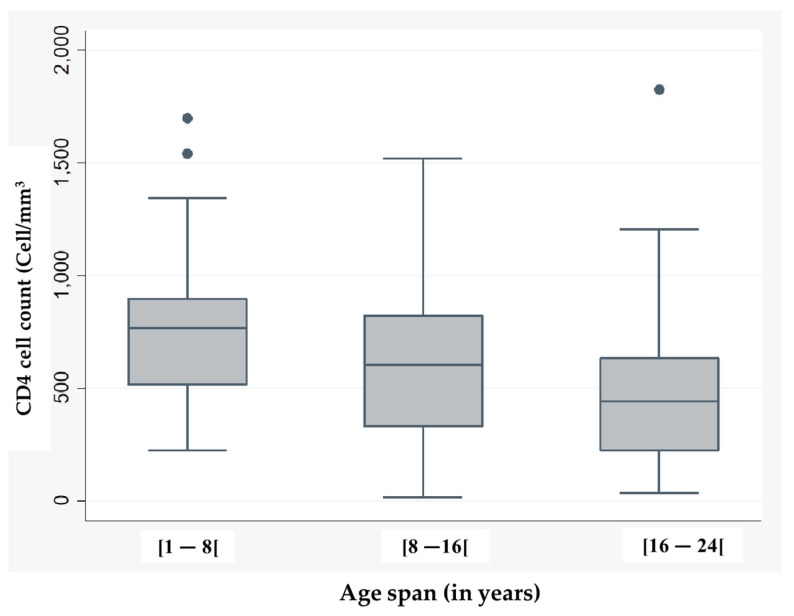
The distribution of CD4 counts across the age span.

The patients’ immunity was assessed by the number of CD4 cells per mm^3^. The median CD4 count was 545, with a minimum of 38 and a maximum of 1825 cells/mm^3^. The immunity level, assessed in three categories (highly immunocompromised, weakly immunocompromised, and immunocompetent), shows that the most highly immunocompromised age span was 16 to 24 years old, followed by the 8 to 15 years old and the 1 to 7 years old (Figure 1).

Across the remaining immune levels (weakly immunocompromised and immunocompetent), age span did not differ significantly in CD4 cell counts (Table 2).

**Figure 2:**
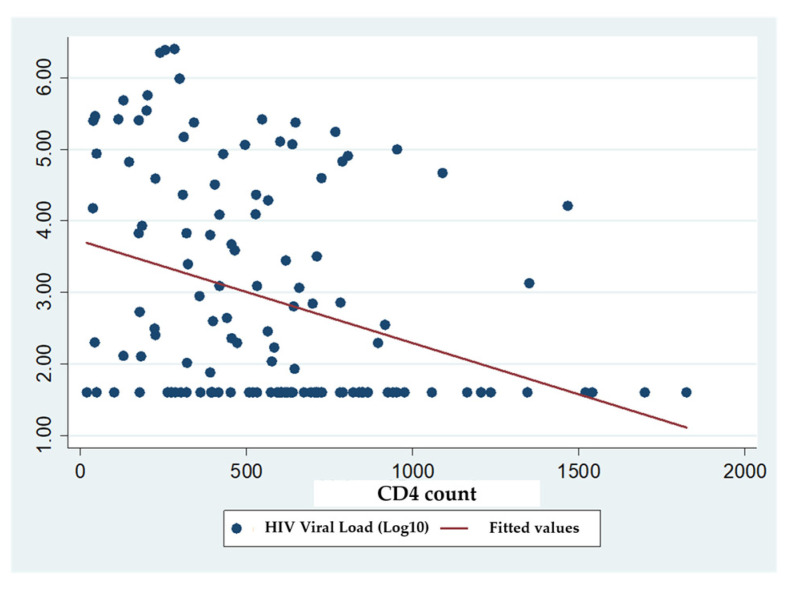
This figure shows the CD4 cell count dispersion according to HIV viral load.

A negative correlation was observed between CD4 cell counts and HIV viral load (r = −0.34; *p* = 0.0001), indicating that elevated CD4 counts were associated with reduced viral loads (Figure 2).

### 3.3. Virological Suppression by Age and Treatment Duration

**Figure 3:**
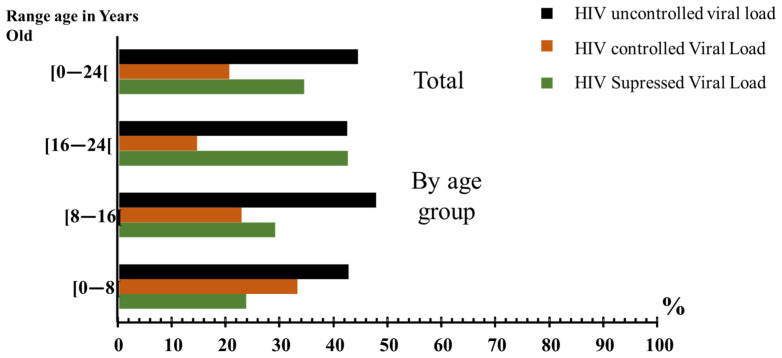
Proportion of patients according to their viral load status and by age group.

The virological analysis showed that 44.6% (58/130) of patients had detectable viral load greater than 1000 copies/mL (HIV uncontrolled viral load). The 8–16 age group had the highest proportion of HIV uncontrolled viral load (47.9%; 23/48), followed by the 0–8 age group (42.86%; 9/21) and the 16–24 age group (42.62%; 26/61) (Figure 3).

Regarding the duration of treatment, more than half of the patients had been treated for over 5 years (40% for those treated for 5 to 10 years and 26.2% for those treated for over 10 years; 66.2% had been treated for more than 5 years). Patients who achieved 5–10 years of therapy showed better suppression rates than those with treatment durations of more than 10 years (Table 3). The percentage of uncontrolled viral load decreased with the treament duration.

## 4. Discussion

Our study reveals that the monitored population is predominantly young, comprising mainly adolescents and young adults. There are slightly more girls, but the data do not allow us to conclude that there is a significant difference between the sexes in terms of viral suppression. The median CD4 cell count indicates a low immune level for adolescents and young adults compared to children. This indicates that this particular population highlights a real problem in the control of HIV infection. This observation is consistent with recent cohort analyses showing that adolescents and young adults living with HIV tend to present lower median CD4 counts compared to children [9,10]. Longitudinal studies highlight that while children often maintain relatively higher CD4 levels under antiretroviral therapy, these values decline progressively during adolescence and early adulthood, reflecting a critical vulnerability in immune control [11]. Clinical guidelines highlight that the transition from pediatric to adolescent care represents a critical period associated with an increased risk of immunological decline, thereby emphasizing the importance of strengthened monitoring and adherence strategies during this stage [11].

In parallel, this study highlights a real challenge in achieving viral suppression among adolescents and young adults living with HIV in Gabon. Although more than half of the participants achieved viral load suppression (<1000 copies/mL), nearly 45% remained uncontrolled, underscoring a significant gap compared to the UNAIDS 95-95-95 target [12]. This gap reflects not only biological vulnerabilities associated with lower median CD4 counts in adolescents and young adults, but also structural and behavioural barriers that hinder effective treatment outcomes [13,14]. Factors such as delayed diagnosis, inconsistent adherence to antiretroviral therapy, and limited access to virological monitoring contribute to the persistence of uncontrolled viral load in this age group [15]. These findings emphasize the urgent need for tailored interventions that address the unique challenges faced during adolescence and early adulthood, including strengthened counselling, peer-support mechanisms, and transition programs from pediatric to adult HIV care. Bridging this gap is essential if Gabon is to align with the UNAIDS 95-95-95 target and ensure durable viral suppression across all age groups.

The lower suppression observed in children aged 0–8 years likely reflects perinatal acquisition with initially higher viremia, reliance on caregiver adherence and clinic attendance, and formulation constraints in pediatric ART. While our analytic emphasis was on adolescents and young adults due to their predominance in care and policy relevance to UNAIDS targets, these pediatric findings highlight a critical need for tailored, child-centred adherence and formulation strategies and warrant dedicated longitudinal investigation [16,17].

Our chi-square test revealed a significant difference in age distribution across immunity categories (global *p* = 0.0263), with late adolescents and young adults (16–24 years) disproportionately represented among highly immunocompromised individuals (70% vs. 57.6% in weakly immunocompromised and 37% in immunocompetent). This pattern, consistent with international cohort analyses [12,18,19], underscores that immunological vulnerability in this age span is a global phenomenon, warranting adolescent-centred strategies to strengthen immune recovery and viral suppression.

The inverse correlation between viral load and CD4 counts observed here aligns with established immune-virological dynamics [20,21,22]. Although viral suppression tends to improve with age, uncontrolled viremia remains common among young adults, reflecting both improved treatment literacy and persistent adherence challenges during the transition from pediatric to adult care. Studies from Kenya and South Africa highlight how disclosure, stigma, and treatment independence contribute to poorer outcomes in adolescents compared to children [23,24,25].

Our study highlights a better viral load suppression rate among patients treated for more than 5 years. That could indicate that sustained exposure to antiretroviral therapy not only enhances treatment literacy and adherence behaviors but also reflects the cumulative benefits of long-term immune reconstitution. Extended treatment duration may reduce the likelihood of resistance development through consistent viral suppression, while also allowing patients to integrate therapy into daily routines, thereby minimizing missed doses. Similar findings have been reported in longitudinal cohorts from Uganda and Tanzania, where longer treatment duration was associated with improved virological outcomes and reduced risk of treatment failure [26,27]. This underscores the importance of early initiation and continuity of care, as durable suppression appears to be strongly linked to both biological recovery and psychosocial adaptation over time.

From a public health perspective, these findings underscore the urgent need for adolescent-focused interventions in Gabon. Strengthening psychosocial support, structured transition services, and differentiated delivery models (including peer support, mobile reminders, and extended refill intervals) could help mitigate the high burden of uncontrolled viral loads in this population. Despite the inherent limitations of our cross-sectional design, this study provides rare evidence from Central Africa and reinforces the importance of tailored medical, psychosocial, and structural strategies to reduce treatment failure and achieve long-term HIV targets.

## 5. Conclusions

In summary, this study highlights the particular vulnerability of adolescents and young adults living with HIV in Gabon, characterised by lower median CD4 counts and suboptimal viral suppression rates. Although prolonged antiretroviral therapy (>5 years) was associated with improved virological outcomes, nearly half of the cohort remained unsuppressed, underscoring persistent biological, behavioural, and structural barriers. These findings emphasise the urgent need for adolescent-centred interventions, including strengthened psychosocial support, structured transition programs, and differentiated service delivery models, while also addressing pediatric-specific challenges such as caregiver adherence and formulation constraints. Despite the limitations of a cross-sectional design, this work provides rare evidence from Central Africa and reinforces the necessity of tailored medical and psychosocial strategies to achieve durable viral suppression and align with the UNAIDS 95-95-95 targets across all age groups.

## Figures and Tables

**Table 1:** Composition of the studied population.

Age Span (Years)	Total	Male	Femele
Total	130	59	71
[0–8[	21	11	10
[8–16[	48	20	28
[16–24[	61	28	33

**Table 2:** Distribution of Immunity Levels by Age span. The table presents, on the left, the three immunity levels of the total population, with the number of individuals and the percentage in brackets. The table displays the three immunity levels and patient proportions by age span. The *p*-value column expresses the result of the chi-square or Fisher’s exact tests.

Immunity Level	N (%)	Age Span (in Years); *n* (%)	*p*-Value
Highly immunocompromised	24 (18.5)	[0; 8 [; 1 (4.2)	*p* < 0.001
		[8; 16 [; 6 (25)	
		[16; 24 [; 17 (70)	
Weakly immunocompromised	33 (25.4)	[0; 8 [; 6 (18.2)	*p* = 0.0028
		[8; 16 [; 8 (24.2)	
		[16; 24 [; 19 (57.6)	
Immunocompetent	73 (56.1)	[0; 8 [; 14 (19.2)	*p* = 0.004
		[8; 16 [; 32 (43.8)	
		[16; 24 [; 27 (37)	

**Table 3:** Treatment duration and viral load status. This table presents the treatment duration and the number of patients (with percentage) in each category of HIV viral load status.

Time of Infection	N (%)	Suppresed VL *n* (%)	Controled VL *n* (%)	Uncontroled VL *n* (%)	*p*-Value
<5 years	44 (33.8)	16 (36.4)	7 (15.9)	21(47.7)	*p* = 0.037
5–10 years	52 (40)	28 (53.9)	5 (9.6)	19 (36.5)	
>10 years	34 (26.2)	14 (41.2)	11 (32.3)	9 (26.5)	

## Data Availability

The raw data supporting the conclusions of this article will be made available by the authors on request.
